# The Characterization of an Efficient Phenylpyruvate Decarboxylase KDC4427, Involved in 2-Phenylethanol and IAA Production from Bacterial Enterobacter sp. CGMCC 5087

**DOI:** 10.1128/spectrum.02660-21

**Published:** 2022-04-04

**Authors:** Wenzhi Bao, Xing Li, Jinfeng Liu, Rong Zheng, Lijuan Liu, Haibo Zhang

**Affiliations:** a Qingdao Institute of Bioenergy and Bioprocess Technology, Chinese Academy of Sciences, Qingdao, China; b Shandong Energy Institute, Qingdao, China; c College of Life Science and Technology, Inner Mongolia Normal University, Hohhot, China; University of Minnesota

**Keywords:** 2-phenylethanol, phenylpyruvate decarboxylase, KDC4427, indolepyruvate decarboxylase

## Abstract

Phenylpyruvate decarboxylase (PPDC) is a crucial enzyme that plays important roles in 2-phenylethanol (2-PE) biosynthesis. In our previous study, we screened a highly efficient PPDC KDC4427 from the novel 2-PE-producing strain Enterobacter sp. CGMCC 5087. Meanwhile, its decarboxylation activity of indolylpyruvate (IPyA) was also higher than other indolylpyruvate decarboxylases (IPDCs) reported so far. In this study, KDC4427 protein was purified and characterized, and its catalytic mechanisms were analyzed by biological methods. The optimum pH and temperature of KDC4427 was pH 6.5 and 35°C, respectively. The enzyme activity was relatively stable between pH 6 and 8 and over the range of temperatures from 25°C to 45°C. KDC4427 showed the highest catalytic efficiency on phenylpyruvic acid (PPA); meanwhile, it also showed high activity for IPyA and 2-ketobutanoic acid, and it was found that KDC4427 belongs to IPDCs by phylogenetic tree analysis. The coverage of the three-dimensional structure of KDC4427 and *Ec*IPDC from Enterobacter cloacae was 96%. Leucine 542, one of the residues in the substrate-binding pocket, is replaced by isoleucine in KDC4427 compared with *Ec*IPDC. Site-directed mutagenesis showed that the transition from leucine to isoleucine was unlikely to make KDC4427 have high catalytic activity for PPA and IPyA; the mutants at glutamate 468 almost completely lost catalytic activities for both PPA and IPyA, indicating that this glutamate was essential for the catalytic activity. Additionally, alanine 387 plays an important role in the substrate selectivity of KDC4427.

**IMPORTANCE** Compared with the chemical synthesis of 2-phenylethanol (2-PE) by condensation of ethylene oxide and benzene, the biological synthesis of 2-PE is a potential method to replace the traditional process. This makes biotransformation gradually become the main way to produce high-quality 2-PE. Phenylpyruvate decarboxylase (PPDC) is the critical enzyme in 2-PE biosynthesis, and it is a momentous point of penetration to increase the production of 2-PE. In this regard, KDC4427 can catalyze phenylpyruvic acid (PPA) to phenylacetaldehyde more efficiently than any other PPDC previously reported. Moreover, it has high activity of indolepyruvate decarboxylases (IPDCs), which will be a great breakthrough in the synthesis of indole-3-acetic acid (IAA). With this study, we offer insights into the KDC4427 catalytic mechanism and significantly expand the toolbox of available α-ketoacid decarboxylases for application in biosynthesis.

## INTRODUCTION

The compound 2-phenylethanol (2-PE), a compound with rose-like aroma and stable physicochemical properties, naturally exists in the flowers and leaves of plants or the essential oils extracted from them (1); it is widely used in food, daily chemical products, and even medicine (2). Furthermore, 2-PE is a significant material in the manufacture of styrene, phenylethylacetate, and other widely used substances (3). The synthetic methods of 2-PE include chemical methods and biological methods. At present, there are two main chemical synthesis methods of 2-PE, one is the hydrogenation of styrene oxide and the other is the synthesis of benzene and ethylene oxide (4, 5). However, there are some defects in the two chemical synthesis methods, such as low purity of products and more byproducts (6). The phenylpyruvate and Ehrlich pathways are the main pathways of 2-PE synthesis in microbes, and phenylpyruvate decarboxylase (PPDC) is the main rate-limiting enzyme (7).

PPDC is an α-ketoacid decarboxylase and is thiamine diphosphate (ThDP) dependent. It catalyzes phenylpyruvic acid (PPA) to produce phenylacetaldehyde and is indispensable in the process of microbial synthesis of 2-PE. It is widely present in plants, fungi, and some bacteria (8–11). At present, only the PPDCs of Saccharomyces cerevisiae and Azospirillum brasilense have been identified for their biochemical functions (12, 13). Aro10 from S. cerevisiae has a wide range of substrate specificity and also catalyzes decarboxylation of indolylpyruvate (IPyA) (13, 14). Indolylpyruvate decarboxylase (IPDC) is the main rate-limiting enzyme in indole-3-acetic acid (IAA) biosynthesis. Among the reported IPDCs, the activity of *Ec*IPDC from Enterobacter cloacae was the highest, and the enzyme characterization and structure analysis were carried out *in vitro* (15). However, PPA was hardly decarboxylated by *Ec*IPDC (16).

In recent years, researchers have heterologously expressed related genes in Escherichia coli, and great progress has been made in the production of 2-PE (11, 17–19), indicating that bacteria have potential applications and research value in 2-PE synthesis. However, there are few studies on the biosynthesis pathway of 2-PE in bacteria, especially the identification and research of PPDC. Therefore, finding novel PPDCs, especially those from bacteria with high activity and specificity, is an important strategy to improve the biosynthesis of 2-PE. Recently, a novel PPDC composed of two subunits was found in Streptomyces virginiae (20). Enterobacter sp. CGMCC 5087, a 2-PE-producing strain, was identified in our previous study (21). The strain was able to *de novo* synthesize 2-PE through the phenylpyruvate pathway. We have previously reported that KDC4427 had PPDC activity, and it was found that the activity of KDC4427 was higher than Aro10 from S. cerevisiae (22). Furthermore, in our unpublished work, we demonstrated that it has higher activity of PPDC and IPDC and is essential for Enterobacter sp. CGMCC 5087 to produce 2-PE and IAA.

Based on the above, this study analyzes the biological characteristics of KDC4427, including enzymatic properties, phylogenetics, substrate specificity, and three-dimensional structure simulation. Furthermore, the catalytic mechanism of KDC4427 was elucidated by site-directed mutagenesis, which provided theoretical guidance for the directed evolution of KDC4427.

## RESULTS

### Expression and purification of KDC4427.

**FIG 1:**
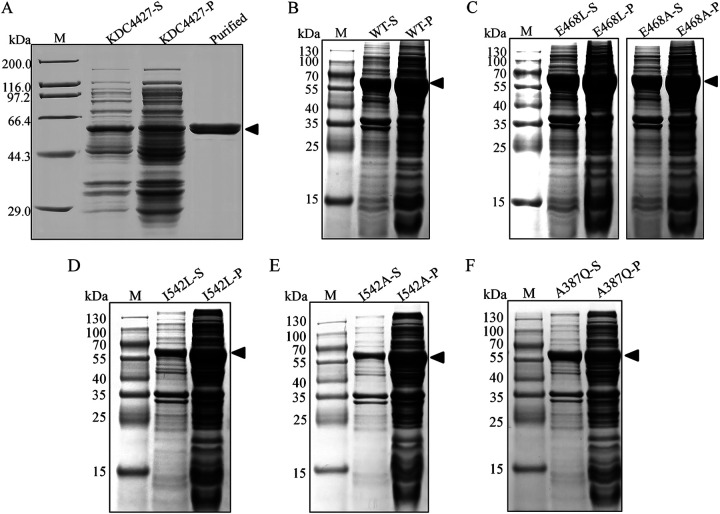
SDS-PAGE was used to verify protein expression and purification. (A) Verification of expression and purification of KDC4427 in BL21(DE3) cells. (B) Expression of wild-type KDC4427 in KDC4427-null strains. (C to F) Expression of KDC4427 mutants in KDC4427-null strains; M, molecular mass of the protein; S, the supernatant after bacterial fragmentation; P, the precipitation after bacterial fragmentation; purified, purified protein.

KDC4427 protein was expressed in BL21(DE3) cells with an N-terminal 6×His tag. The soluble protein was detected by sodium dodecyl sulfate-polyacrylamide gel electrophoresis (SDS-PAGE). According to the detection results, KDC4427 is well expressed in the bacteria, and almost half of the protein is in soluble form in the cell-free fraction, which is conducive to the purification of the protein (Fig. 1A). The band in lane 3 represents the purified KDC4427; it can be seen that the purity of KDC4427 reaches the standard of enzyme activity reaction (Fig. 1A). On the basis of SDS-PAGE, the molecular mass of KDC4427 is approximately 62.7 kDa, corresponding well to the one expected. The concentration of purified KDC4427 was determined by a bicinchoninic acid (BCA) protein quantitative kit, and the standard curve was drawn. The measured protein concentration was 0.9 mg/mL.

### Effects of pH and temperature on enzyme activity.

**FIG 2:**
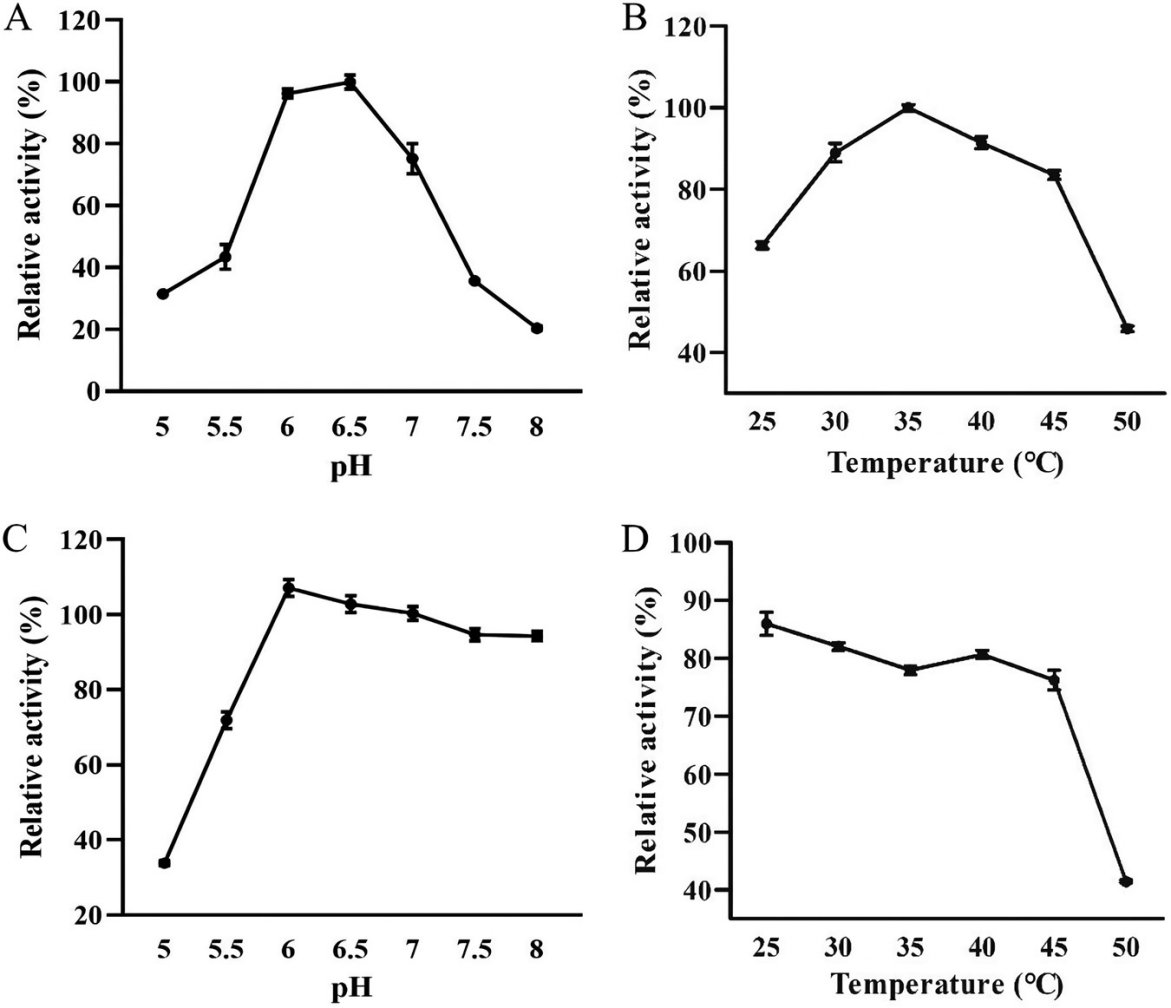
Enzymatic properties of KDC4427. (A) Determination of the optimum pH of KDC4427. (B) Determination of the optimum temperature of KDC4427. (C) Stability of KDC4427 protein under different pH conditions. (D) Stability of KDC4427 protein at different temperatures. The stability of the enzyme was determined after incubation for 1 h under different conditions. Each point represents the mean ± standard error of the mean (SEM) of three independent test results.

To investigate the optimum pH and temperature of KDC4427, the activity and stability of KDC4427 were tested at different pH values and temperatures. As can be seen from Fig. 2A, the activity of KDC4427 is the highest at pH 6 to 6.5. When the pH was lower than 5 or higher than 8, the enzyme activity was only 30% and 20% of the maximum activity, respectively. This is consistent with the characteristics of ThDP-dependent enzymes (23). The effect of temperature on KDC4427 enzyme activity is shown in Fig. 2B. The optimum temperature was 37°C, and when the temperature was below 25°C or above 50°C, the enzyme activity was only 70% and 30% of the highest activity.

KDC4427 was relatively stable between pH 6 and 8, and the activity of KDC4427 decreased significantly when the pH was lower than 6.0 (Fig. 2C). When the pH was 5.0, the residual enzyme activity was only 33.8%. Over the range of temperatures from 25°C to 50°C, the thermostability of purified KDC4427 was tested. The enzyme activity was stable when the temperature was below 45°C and gradually decreased when the temperature rose to 45°C. Only 41% of the residual activity was detected after incubation at 50°C for 1 h (Fig. 2D).

### Comparative analysis of physicochemical properties of amino acids from KDC4427, Aro10, and *Ec*IPDC.

Isoelectric point (pI) is the pH value of a protein or a specific molecule without net charge (electronegativity). The amino acid sequence of KDC4427 has more negatively charged residues than positively charged residues, which makes the enzyme weakly acidic (pI < 7.0); this may be the main reason for the high activity of KDC4427 in weak acid environments. Both *Ec*IPDC and Aro10 are also weakly acidic, but the pI value of *Ec*IPDC is closer to KDC4427 (Table 1). Instability index (II) is used to measure the stability of protein (24). The II values of the three enzymes were less than 40, indicating that the enzyme was stable under natural conditions. The relative volume of aliphatic side chains in protein is defined as aliphatic index (AI), which represents the thermal stability of protein (24). The AI value of KDC4427 is 93.38, which indicates that KDC4427 contains more fatty acid side chains, which may be one of the main factors affecting the temperature adaptability of KDC4427. The AI value of *Ec*IPDC was only 1.37 lower than that of KDC4427, while that of Aro10 was 4.37 lower, indicating that Aro10 had relatively fewer fatty acid side chains. The grand average of hydropathicity (GRAVY) reflects the interaction between protein and water (24), and the GRAVY values in the sequences were less than 0, indicating that KDC4427, *Ec*IPDC, and Aro10 are hydrophilic proteins, and Aro10 is more hydrophilic.

**TABLE 1:** Physicochemical properties of amino acids

Source	Asp + Glu	Arg + Lys	pI^*a*^	II^*b*^	AI^*c*^	GRAVY^*d*^
KDC4427	59	43	5.73	37.28	93.38	−0.030
*Ec*IPDC	59	42	5.67	38.29	92.01	−0.017
Aro10	70	63	6.10	33.61	89.01	−0.216

a pI, isoelectric point.

b II, instability index.

c AI, aliphatic index.

d GRAVY, grand average of hydropathicity.

### Activity and substrate specificity analysis.

In order to investigate the activity and substrate specificity of KDC4427, the purified protein was reacted with different substrates, and the catalytic efficiency of different substrates was measured and calculated. As shown in Table 2, PPA is the preferred substrate for KDC4427, followed by 2-ketobutanoic acid and IPyA. According to the *K*_m_ value, IPyA has the highest affinity for KDC4427, although the decarboxylation is more slow than PPA and 2-ketobutanoic acid; 2-ketobutanoic acid, the substrate derived from glutamate, had a similar affinity and decarboxylation efficiency as PPA for KDC4427. The substrate 2-ketopentanoic acid had approximately 40-fold lower affinity than IPyA but was decarboxylated more quickly, with a *k*_cat_ value approaching 130 s^−1^. Similar results were obtained for the substrate 2-ketohexanoic acid. The affinity of 3-methyl-2-ketobutanoic acid derived from valine was twice that of PPA, but its *k*_cat_ value was much lower than that of PPA. In contrast, pyruvic acid from alanine was a much worse substrate for KDC4427. In general, these data indicated that the substrates derived from aromatic amino acids were significantly better than those of aliphatic amino acids and confirmed that the *KDC4427* gene product was an effective aromatic α-ketoacid decarboxylase.

**TABLE 2:** Substrate specificity and kinetic parameters of KDC4427

Substrates	*K*_m_ (mM)^*a*^	*k*_cat_ (s^−1^)^*a*^	*k*_cat_/*K*_m_ (mM^−1^ s^−1^)	Amino acid	%^*b*^
Phenylpyruvic acid^*c*^	0.60 ± 0.02	186.94 ± 0.39	311.76	Phe	100
2-Ketobutanoic acid	0.66 ± 0.08	185.77 ± 3.08	255.09	Glu	81.82
Indole-3-pyruvic acid^*c*^	0.015 ± 0.002	3.7 ± 0.6	247	Trp	79.23
2-Ketopentanoic acid	0.65 ± 0.02	128.77 ± 2.35	198		63.51
2-Ketohexanoic acid	0.52 ± 0.01	78.84 ± 0.51	152.25		48.84
3-Methyl-2-ketobutanoic acid	0.31 ± 0.07	34.88 ± 1.86	115.55	Val	37.06
3-Methyl-2-ketopentanoic acid	0.72 ± 0.06	29.42 ± 0.86	40.95	Ile	13.14
Pyruvic acid	0.86 ± 0.15	22.31 ± 2.79	26.1	Ala	8.37

a All data represent mean ± standard deviation of three replicates.

b *k*_cat_/*K*_m_ of phenylpyruvate was taken as the percentage.

c The data of PPA and IPyA were cited from our unpublished paper.

### Phylogenetic analysis.

**FIG 3:**
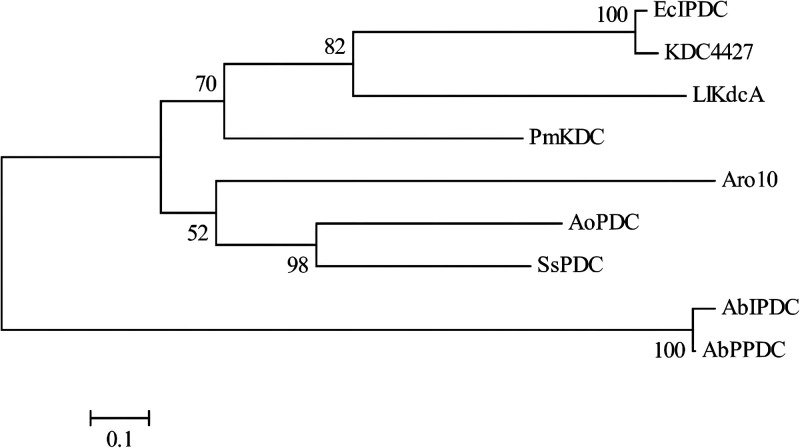
Phylogenetic tree of KDC4427. Accession numbers are shown in Table S2 in the supplemental material. The amino acid sequences of the above enzymes were compared by Mega 6 software, and the *Ec*IPDC closest relatives with KDC4427 was used as the homologous model for the following research.

According to the activity characteristics of KDC4427, it can be seen that it has high decarboxylation efficiency for PPA and IpyA. Therefore, phylogenetic tree analysis of KDC4427 and reported α-ketoacid decarboxylase was carried out. Phylogenetic tree analysis showed that the closest genetic relationship with KDC4427 was the *Ec*IPDC of E. cloacae; the amino acid identity was 94% (Fig. 3). It can be seen that KDC4427 belongs to IPDCs. The amino acid similarity between KDC4427 and Aro10 of S. cerevisiae was only 31%. The α-ketoacid decarboxylase with the farthest relationship to KDC4427 was *Ab*PPDC of *A. brasiliense*; the amino acid identity was 29%.

### Sequence homology analysis and homology modeling.

**FIG 4:**
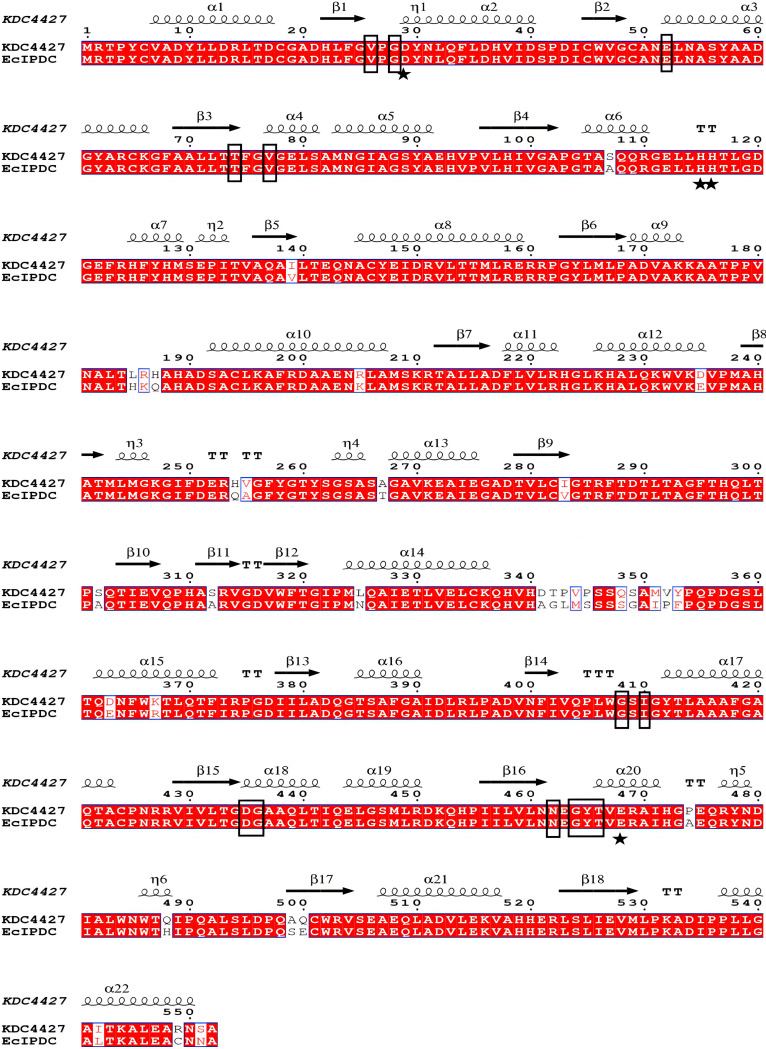
Amino acid sequence comparison of KDC4427 and *Ec*IPDC. Amino acids with black borders represent ThDP-binding sites. The “HH-motif” is marked with a black asterisk.

**FIG 5:**
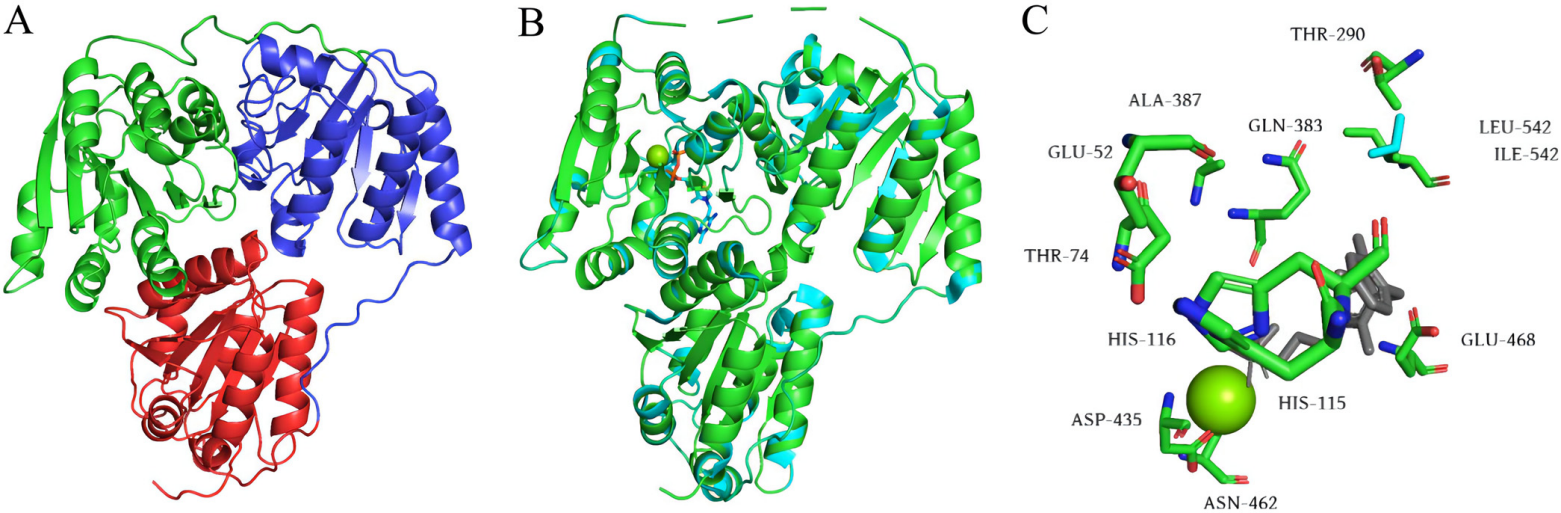
Prediction and analysis of KDC4427 tertiary structure. (A) KDC4427 homology model. The PYR (N-terminal) domain, PP (C-terminal) domain, and middle domain are shown in red, green, and blue, respectively. (B) Superposition of the tertiary structure of KDC4427 (green) and *Ec*IPDC (cyan; PDB: 1OVM). (C) Superposition of active sites between KDC4427 (green) and *Ec*IPDC (cyan).

In order to predict the key amino acid residues of KDC4427, the amino acid sequence of KDC4427 was compared with that of *Ec*IPDC (Fig. 4). The α-helix and random coil are the main secondary structural elements, in which random coil accounts for 40%, α-helix accounts for 37.64%, extended strand accounts for 17%, and β-fold accounts for 5.09% of proportions. Sequence alignment showed that KDC4427 had the same ThDP-binding site as *Ec*IPDC and also had two successive histidines in one peptide chain, which is called an “HH-motif.” In the three-dimensional structure simulation, KDC4427 and *Ec*IPDC have a high coincidence degree (the coverage rate is 96%, and the confidence is 100%) of stereostructure and generally formed a tetramer and had two ligands, ThDP and Mg^2+^. This is confirmed by the homologous model built with the phyre v2.0 server (Fig. 5A). The KDC4427 monomer model consists of three domains: the PYR domain (residues 3 to 180), intermediate domain (residues 181 to 340), and PP domain (residues 356 to 551). The N terminus and C terminus are in the pyrimidine (PYR) and pyrophosphate (PP) domains, respectively. The domains PYR and PP are responsible for binding to ThDP and the phosphoric acid parts of some cofactors, respectively. In the superposition of stereostructure, the difference between KDC4427 (green) and *Ec*IPDC (cyan) was concentrated mainly in the middle domain and the middle part with the PP domain (Fig. 5B). In the analysis of binding sites, the 542 isoleucine of KDC4427 replaces the leucine of *Ec*IPDC (Fig. 5C), which may be responsible for the different substrate specificity of KDC4427.

### Detection of KDC4427 mutant activity.

The putative binding site amino acid residues I542 and A387 and catalytic residue E468 were used to investigate the function of these residues in KDC4427 catalysis. KDC4427 and mutants I542A, I542L, A387Q, E468A, and E468L were expressed *in vivo*, and the activities were examined. As shown in Fig. 1, the wild-type KDC4427 and all the mutants were expressed well, and the molecular masses observed on SDS-PAGE gels were consistent with the expectation.

**FIG 6:**
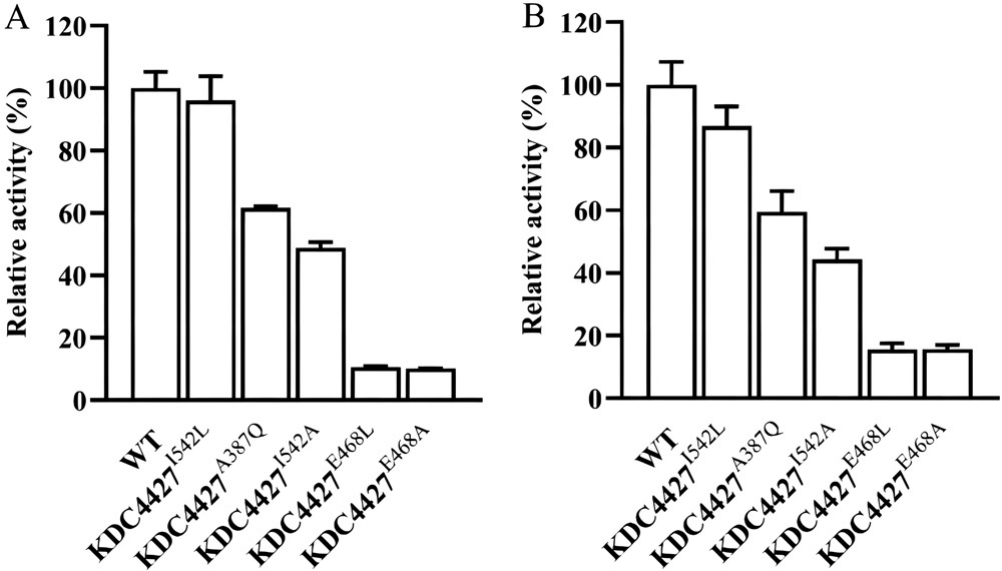
Activity detection of KDC4427 mutants. (A) The production of IAA in various mutants cultured for 24 h. (B) The production of 2-PE in various mutants cultured for 24 h. After the mutants were cultured at 37°C and 200 rpm for 24 h, the levels of secreted IAA and 2-PE were determined. Error bars indicate standard deviation (*n* = 3).

The substrate-binding sites of *Ec*IPDC were the Thr-Ala-Leu triad (15), and the corresponding amino acids of KDC4427 were Thr-Ala-Ile. In order to confirm the function of Ile542 in KDC4427, I542A and I542L mutants were constructed. In *Ec*IPDC, amino acid residue L542 increased the hydrophobicity of the binding cavity, thus stabilizing the zwitterionic intermediate (15). In KDC4427, the activity of mutant I542L for IPyA is almost unchanged, indicating that the high IPyA activity of KDC4427 is not due to the transition of I542 (Fig. 6A). Although, the decrease of decarboxylation activity of KDC4427 for PPA is higher than IPyA, KDC4427 still has more than 80% enzyme activity, which is enough to show that the replacement of I542 is not the major associated reason leading to its PPDC activity (Fig. 6B). When I542 was mutated to alanine, the enzyme activity decreased about 60%, indicating that I542 was essential in substrate binding. Isoleucine is also a hydrophobic amino acid, which can increase the hydrophobicity of the active site, which is in line with the above conclusion. A387 is one of the amino acids that affects substrate specificity in *Ec*IPDC (25). After A387 was mutated to glutamate, the catalytic activities of PPA and IPyA decreased 40%, respectively, suggesting that A387Q had the same selectivity to the above two substrates, and the reason for the decrease of activity might be that glutamine makes the substrate-binding cavity narrow. The Asp-His-Glu catalytic domain is the conserved catalytic active site of the “HH-motif” decarboxylase (25). Among them, only leucine or alanine substituted glutamic acid (26, 27) in the catalytic triad of *Ab*PPDC. The corresponding catalytic site of KDC4427 is still Asp-His-Glu. In order to confirm whether Glu468 is the main amino acid affecting the decarboxylation activity of KDC4427, mutants E468A and E468L were constructed, and their activities were determined. The mutation of E468L did not increase the decarboxylation activity of PPA but almost completely lost the two catalytic activities, as did the mutant E468A. This result demonstrated that E468 plays a key role in the catalysis of KDC4427.

## DISCUSSION

In recent years, biological synthesis of 2-PE has attracted extensive attention of researchers worldwide (2). It has been reported that 2-PE was produced by heterologous expression of related genes in E. coli (19). This indicates that bacteria have potential application and research value in 2-PE synthesis. PPDC is a rate-limiting enzyme in the biosynthesis of 2-PE. KDC4427 is a PPDC with high activity from Enterobacter sp. CGMCC 5087. In this study, we characterized the enzymatic properties of KDC4427, and the substrate specificity was analyzed by enzyme activity assay. Meanwhile, the catalytic mechanism of KDC4427 was further clarified by sequence alignment, structural simulation, and site-directed mutation.

The activity of KDC4427 was the highest at pH 6.5 and decreased sharply at pH 7 to 7.5. This may be due to the pH-dependent association of KDC4427 subunits. In previous reports, *Ec*IPDC existed mainly in the form of a tetramer at pH 5.6 to 6.0 and rapidly dissociated into a dimer at pH 6.7 to 7.4 (28). In the presence of ThDP and Mg^2+^ (cofactors) in the buffer, *Ec*IPDC was stable at 50°C after incubation for 30 min (16), while KDC4427 had low stability and only 40% of activity after incubation for 1 h without cofactors (Fig. 2D); it appears that the presence of cofactors may improve the thermal denaturation resistance of KDC4427. According to the amino acid AI value (Table 1), it can be inferred that the thermal stability of KDC4427 with cofactors is stronger than that of *Ec*IPDC. The residual activity of previously reported *Pm*KDC, a ThDP-dependent α-ketoacid decarboxylase from *Proteus mirabilis* JN458, at 25°C is about 30% (29), while KDC4427 has about 70% residual activity (Fig. 2B). At the same time, the stability of KDC4427 at 25°C is more than 85% of activity (Fig. 2D); it can be seen that KDC4427 is an enzyme with high adaptability to low temperature. Low temperature-adapted enzymes are a promising source for the pharmaceutical, agricultural, and chemical industries (30) because the biosynthesis of vulnerable pharmaceutical compounds requires low temperature conditions to avoid side effects and save energy (31).

According to the current data analysis, as a PPDC, KDC4427 has higher catalytic efficiency for a variety of substrates than Aro10. The affinity of Aro10 for PPA is 6-fold higher than that of KDC4427, and the *k*_cat_ value of KDC4427 for PPA is 9-fold higher than that of Aro10 (Table S3 in the supplemental material). Moreover, KDC4427 showed a relatively higher catalytic efficiency for PPA than *Ab*PPDC. These data suggested that KDC4427 is the PPDC with the highest catalytic activity so far. For KDC4427, as an IPDC, in the reaction with IPyA, the affinity to the substrate was 2-fold higher than that of Aro10 and 0.25-fold higher than that of *Ec*IPDC (Table S3); it has the highest indole pyruvate decarboxylation activity. *Ec*IPDC does not have PPDC activity, although KDC4427 has high homology with it.

The phylogenetic tree showed that KDC4427 was closely related to *Ec*IPDC from E. cloacae and belongs to IPDCs. This confirmed that KDC4427 belongs to both PPDCs and IPDCs and also has these two catalytic activities. The primary cause of this phenomenon may be the distinctive tertiary structure or amino acid sequence. It was found that KDC4427 and *Ec*IPDC had very high similarity, which was unprecedented in the previous report of α-ketoacid decarboxylase. However, it was found that the intermediate domain (also known as the TH3 domain) has a huge difference, which is consistent with the overall characteristics of ThDP-dependent decarboxylases (32). In addition, KDC4427 has two histidine residues on the same polypeptide chain, which is called the “HH-motif.” The enzymes containing HH-motifs include pyruvate decarboxylase, IPDC, PPDC, and branched α-ketoacid decarboxylase. In addition to two histidine residues, these enzymes also contain a completely conservative aspartic acid residue and a glutamate residue (28), which is exactly the same as KDC4427. The X-ray structure of a pyruvate decarboxylase mutant from S. cerevisiae and pyruvate complex showed that there was a hydrogen bond between pyruvate decarboxylase and Glu477 of “HH-motif” (33). This report supports the fact that the mutants E468L and E468A almost completely lost their decarboxylation activity in this study. The difference of tetramer packing, the exchange of several amino acids in the substrate-binding pocket, and the varied structure of C-terminal helix covering the active site are regarded as the reasons for the different specificity of α-ketoacid decarboxylase (28). I542 serves as the only different amino acid in binding sites between KDC4427 and *Ec*IPDC; I542 has no significant change in the catalytic activity of IPyA and PPA after being mutated to leucine. Therefore, I542 is not the main reason for which KDC4427 has both phenylpyruvate decarboxylation activity and indole pyruvate decarboxylation activity. Perhaps the answer can be found in the intermediate domain or diverse different construction of the C-terminal helix.

In conclusion, among the reported α-ketoacid decarboxylases, KDC4427 has the highest catalytic activities of PPDC and IPDC, and the catalytic mechanism is similar to *Ec*IPDC. Through the above work and research, we have a more comprehensive understanding of the function of this enzyme, which provides scientific data for expanding understanding of the molecular mechanism of 2-PE synthesis, provides theoretical guidance for the directed evolution of PPDC, and provides a solid foundation for promoting the biosynthesis of 2-PE.

## MATERIALS AND METHODS

### Strains and culture.

*E. coli* DH5α was used to construct and amplify vectors, and *E. coli* BL21(DE3) was used to express recombinant proteins (22). Strains were grown in Luria-Bertani (LB) medium containing 50 μg/mL ampicillin at 37°C and 200 rpm.

Enterobacter sp. CGMCC5087-*ΔKDC4427* (Table S1 in the supplemental material) was cultured in LB medium with spectinomycin at a final concentration of 100 μg/mL. The strains that express *KDC442*7 and *KDC4427* mutants in *KDC4427*-null strains were cultured at 37°C and 200 rpm.

### Prokaryotic expression and purification.

The expressed strain that BL21(DE3) containing the expression plasmid pETDuet-1-*KDC4427* was cultured in 5 mL of liquid LB medium with ampicillin antibiotics at 37°C and 200 rpm for 12 h. Three milliliters of culture broth was inoculated into 100 mL of liquid LB medium and grown at 37°C until it reached a turbidity (600 nm) of 0.6 (2 to 3 h). After isopropyl-β-d-thiogalactopyranoside (IPTG) was added to induce protein expression, the supernatant was extracted and purified by nickel-nitrilotriacetic acid (Ni-NTA) His bind resin (Novagen) column. The column was washed with binding buffer (50 mM NaH_2_PO_4_, 300 mM NaCl, and 10 mM imidazole) to balance the column. The impure proteins were washed away with wash buffer (20 mM imidazole and 8% glycerol), and the eluent was reacted with G250 to verify whether the impure protein was washed clean. Elution buffer (250 mM imidazole and 10% glycerol) was used to elute the protein. The purity and molecular mass of the protein were verified by polyacrylamide gel electrophoresis. The purified KDC4427 protein was determined by the BCA (Solarbio, China, Beijing) standard curve method for determination of protein concentration.

### Enzymatic assays.

To characterize the effect of different pH values on the activity of KDC4427, the pH was varied from 5 to 8 by using sodium citrate, sodium phosphate buffers. The 1-mL reaction system (0.2 mM ThDP, 0.1 mM MgSO_4_, and 24 μg/mL KDC4427) was prepared in buffer solution with different pH values, and 2 mM PPA was added to start the reaction. The mixture was incubated for 30 min at 37°C. The reaction products were filtered with 2-μm sterile membranes and then divided into liquid-phase vials. The detection method of phenylacetaldehyde by high-performance liquid chromatography (HPLC) refers to the content previously reported (22). Authentic phenylacetaldehyde (Aladdin, China, Shanghai) was used as the standard. In the determination of the optimum temperature of enzyme activity, the reaction system was prepared in phosphate buffer solution at pH 7.0. The reaction was carried out at 25°C, 30°C, 35°C, 40°C, 45°C, and 50°C for 30 min, and then the enzyme activity was determined as described above.

### Enzyme stability.

The purified protein was incubated under different pH values (5.0, 5.5, 6.0, 6.5, 7.0, 7.5, and 8.0) and temperatures (25°C, 30°C, 35°C, 40°C, 45°C, and 50°C) for 1 h, respectively. Then, 0.2 mM ThDP, 0.1 mM MgSO_4_, and 2 mM PPA were added successively, and the reaction time was 30 min at 35°C and pH 6.5. The activity of KDC4427 at the optimum pH and temperature was used as a blank control, and the determination method was the same as above. The residual activity was calculated with the blank control as 100%.

### Substrate specificity and kinetics.

The decarboxylation activity of KDC4427 to different substrates was monitored by the coupling analysis described previously (34). Preparation of the enzyme reaction system (1 mL) included 20 mM sodium phosphate buffer (pH 7.0), 0.35 mM NADH, 0.2 mM thiamine pyrophosphate, 0.1 mM MgSO_4_, 1 U/mL yeast alcohol dehydrogenase, 2 mM PPA, and 24 μg/mL KDC4427 purified protein. The reaction time was 15 min at 37°C. The absorption peak of NADH was detected at 340 nm (reaction of IPyA as the substrate needs to be determined at 366 nm) by an enzyme labeling instrument, and then the PPA activity of KDC4427 was calculated. The catalytic activity of KDC4427 to various substrates was determined according to the same detection principle.

Under standard conditions, different concentrations of PPA were used for the enzyme reaction. The Michaelis-Menten curve was drawn by nonlinear fitting, and the *K*_m_ and *k*_cat_ values were calculated.

### Analysis of physicochemical properties of KDC4427 amino acids.

The physicochemical properties of KDC4427 amino acids were analyzed by using the website ExPASy-ProtParam tool.

### Phylogenetic analysis.

Nine genomic sequences of α-ketoacid decarboxylase were downloaded from GenBank. The phylogenetic tree of KDC4427 was constructed by the Mega 6 program. The similarity of amino acid sequences was compared by the NCBI website.

### Sequence homology analysis and homology modeling.

The sequence homology of KDC4427 and *Ec*IPDC was analyzed by using the website https://espript.ibcp.fr/ESPript/cgi-bin/ESPript.cgi.

Using indole-3-pyruvate decarboxylase (Protein Data Bank [PDB]: 1OVM) as the template, the homology modeling of KDC4427 was carried out online by using the website protein psychology/analysis recognition enginev 2.0 (http://www.sbg.bio.ic.ac.uk/phyre2/html/page.cgi?id=index).

### Cloning and site-directed mutagenesis.

The *KDC4427* gene was amplified with 4427-*Spe* I-F and 4427-*Eco*R I-R primers (Table S4) using pETDuet-1-KDC4427 vector as the template. After the pTargetF plasmid (35) was double digested with *Eco*R I and *Spe* I, the *KDC4427* gene was ligated with a ClonExpress II one-step cloning kit (Vazyme, China, Nanjing). The ligation product was transformed into DH5α competent cells by electrotransformation, and the positive recombinants were screened by spectinomycin resistance. Using pTargetF-KDC4427 plasmid as the template, primers with mutated amino acid sites (Table S4) were used to amplify it. The PCR products were digested with *Dpn* I to eliminate background pTargetF plasmid. Then, the digestion products were transformed into KDC4427-knockout competent cells and verified by sequencing to obtain mutants I542A, I542L, A387Q, E468A, and E468L.

### Assay of mutant activity.

The wild-type Enterobacter sp. CGMCC 5087 and mutant strains with an optical density at 600 nm (OD_600_) of 0.1 were cultured at 37°C and 200 rpm for 24 h, and then 500 μL of bacterial solution was added with equal volume of *n*-heptane to extract 2-PE. Then, the extracted bacterial solution was filtered and analyzed by gas chromatography (GC) to quantify the concentration of 2-PE. The method of 2-PE detection by GC was slightly adjusted on the basis of a previous report (23). The standard curve was drawn, and sample concentration was calculated by using a group of 2-PE standard samples with different concentrations.

The wild-type Enterobacter sp. CGMCC 5087 and mutant strains with an OD_600_ of 0.1 were cultured at 37°C and 200 rpm for 24 h. The yield of IAA was determined by Salkowski’s method as follows (36). Bacterial solution (300 μL) was centrifuged at 13,000 rpm for 3 min, and 200 μL of supernatant was mixed with 400 μL of Salkowski reagent and incubated in the dark at room temperature for 25 min. Then, the absorbance value was detected at 530 nm by a microplate reader. The standard curve was drawn with different concentrations of IAA.

### Data availability.

The sequence of the KDC4427 gene has been deposited in the GenBank database under accession number PWI79650.1.

## References

[1] Qian X, Yan W, Zhang W, Dong W, Ma J, Ochsenreither K, Jiang M, Xin F. 2019. Current status and perspectives of 2-phenylethanol production through biological processes. Crit Rev Biotechnol 39:235–248. doi:10.1080/07388551.2018.1530634. 30570367

[2] Hua D, Xu P. 2011. Recent advances in biotechnological production of 2-phenylethanol. Biotechnol Adv 29:654–660. doi:10.1016/j.biotechadv.2011.05.001. 21601630

[3] Etschmann MM, Bluemke W, Sell D, Schrader J. 2002. Biotechnological production of 2-phenylethanol. Appl Microbiol Biotechnol 59:1–8. doi:10.1007/s00253-002-0992-x. 12073125

[4] Bedoukian PZ. 1967. Perfumery and flavoring synthetics. Biochemistry 24:5907–5918.

[5] Yamaguchi K, Ebitani K, Kaneda K. 1999. Hydrotalcite-catalyzed epoxidation of olefins using hydrogen peroxide and amide compounds. J Org Chem 64:2966–2968. doi:10.1021/jo982347e. 11674379

[6] Keasling JD, Chou H. 2008. Metabolic engineering delivers next-generation biofuels. Nat Biotechnol 26:298–299. doi:10.1038/nbt0308-298. 18327240

[7] Wang Y, Zhang H, Lu X, Zong H, Zhuge B. 2019. Advances in 2-phenylethanol production from engineered microorganisms. Biotechnol Adv 37:403–409. doi:10.1016/j.biotechadv.2019.02.005. 30768954

[8] Liu J, Bai Y, Fan TP, Zheng XH, Cai YJ. 2020. Unveiling the multipath biosynthesis mechanism of 2-phenylethanol in *Proteus mirabilis*. J Agric Food Chem 68:7684–7690. doi:10.1021/acs.jafc.0c02918. 32608230

[9] Dhandapani S, Jin J, Sridhar V, Chua NH, Jang IC. 2019. CYP79D73 participates in biosynthesis of floral scent compound 2-phenylethanol in *Plumeria rubra*. Plant Physiol 180:171–184. doi:10.1104/pp.19.00098. 30804010 PMC6501094

[10] Pan X, Qi H, Mu L, Wen J, Jia X. 2014. Comparative metabolomic-based metabolic mechanism hypothesis for microbial mixed cultures utilizing cane molasses wastewater for higher 2-phenylethanol production. J Agric Food Chem 62:9927–9935. doi:10.1021/jf502239d. 25199087

[11] Liu J, Jiang J, Bai Y, Fan TP, Zhao Y, Zheng X, Cai Y. 2018. Mimicking a new 2-phenylethanol production pathway from *Proteus mirabilis* JN458 in *Escherichia coli*. J Agric Food Chem 66:3498–3504. doi:10.1021/acs.jafc.8b00627. 29560727

[12] Spaepen S, Versées W, Gocke D, Pohl M, Steyaert J, Vanderleyden J. 2007. Characterization of phenylpyruvate decarboxylase, involved in auxin production of *Azospirillum brasilense*. J Bacteriol 189:7626–7633. doi:10.1128/JB.00830-07. 17766418 PMC2168738

[13] Kneen MM, Stan R, Yep A, Tyler RP, Saehuan C, McLeish MJ. 2011. Characterization of a thiamin diphosphate-dependent phenylpyruvate decarboxylase from *Saccharomyces cerevisiae*. FEBS J 278:1842–1853. doi:10.1111/j.1742-4658.2011.08103.x. 21501384

[14] Vuralhan Z, Luttik MA, Tai SL, Boer VM, Morais MA, Schipper D, Almering MJ, Kötter P, Dickinson JR, Daran JM, Pronk JT. 2005. Physiological characterization of the ARO10-dependent, broad-substrate-specificity 2-oxo acid decarboxylase activity of *Saccharomyces cerevisiae*. Appl Environ Microbiol 71:3276–3284. doi:10.1128/AEM.71.6.3276-3284.2005. 15933030 PMC1151862

[15] Schütz A, Sandalova T, Ricagno S, Hübner G, König S, Schneider G. 2003. Crystal structure of thiamindiphosphate-dependent indolepyruvate decarboxylase from *Enterobacter cloacae*, an enzyme involved in the biosynthesis of the plant hormone indole-3-acetic acid. Eur J Biochem 270:2312–2321. doi:10.1046/j.1432-1033.2003.03601.x. 12752451

[16] Koga J, Adachi T, Hidaka H. 1992. Purification and characterization of indolepyruvate decarboxylase. A novel enzyme for indole-3-acetic acid biosynthesis in *Enterobacter cloacae*. J Biol Chem 267:15823–15828. doi:10.1016/S0021-9258(19)49609-9. 1639814

[17] Atsumi S, Hanai T, Liao JC. 2008. Non-fermentative pathways for synthesis of branched-chain higher alcohols as biofuels. Nature 451:86–89. doi:10.1038/nature06450. 18172501

[18] Koma D, Yamanaka H, Moriyoshi K, Ohmoto T, Sakai K. 2012. Production of aromatic compounds by metabolically engineered *Escherichia coli* with an expanded shikimate pathway. Appl Environ Microbiol 78:6203–6216. doi:10.1128/AEM.01148-12. 22752168 PMC3416637

[19] Achmon Y, Ben-Barak Zelas Z, Fishman A. 2014. Cloning Rosa hybrid phenylacetaldehyde synthase for the production of 2-phenylethanol in a whole cell *Escherichia coli* system. Appl Microbiol Biotechnol 98:3603–3611. doi:10.1007/s00253-013-5269-z. 24081322

[20] Xu XS, Wang C, Chen J, Yang S. 2017. *Streptomyces virginiae* PPDC is a new type of phenylpyruvate decarboxylase composed of two subunits. ACS Chem Biol 12:2008–2014. doi:10.1021/acschembio.7b00307. 28719183

[21] Zhang H, Cao M, Jiang X, Zou H, Wang C, Xu X, Xian M. 2014. *De-novo* synthesis of 2-phenylethanol by *Enterobacter* sp. CGMCC 5087. BMC Biotechnol 14:30. doi:10.1186/1472-6750-14-30. 24766677 PMC4005845

[22] Liu C, Zhang K, Cao W, Zhang G, Chen G, Yang H, Wang Q, Liu H, Xian M, Zhang H. 2018. Genome mining of 2-phenylethanol biosynthetic genes from *Enterobacter* sp. CGMCC 5087 and heterologous overproduction in *Escherichia coli*. Biotechnol Biofuels 11:305. doi:10.1186/s13068-018-1297-3. 30455734 PMC6223000

[23] Nemeria N, Korotchkina L, McLeish MJ, Kenyon GL, Patel MS, Jordan F. 2007. Elucidation of the chemistry of enzyme-bound thiamin diphosphate prior to substrate binding: defining internal equilibria among tautomeric and ionization states. Biochemistry 46:10739–10744. doi:10.1021/bi700838q. 17715948

[24] Faya N, Penkler DL, Tastan Bishop Ö. 2015. Human, vector and parasite Hsp90 proteins: a comparative bioinformatics analysis. FEBS Open Bio 5:916–927. doi:10.1016/j.fob.2015.11.003. PMC4688443 26793431

[25] Andrews FH, McLeish MJ. 2012. Substrate specificity in thiamin diphosphate-dependent decarboxylases. Bioorg Chem 43:26–36. doi:10.1016/j.bioorg.2011.12.001. 22245019

[26] Versées W, Spaepen S, Vanderleyden J, Steyaert J. 2007. The crystal structure of phenylpyruvate decarboxylase from *Azospirillum brasilense* at 1.5 Å resolution. Implications for its catalytic and regulatory mechanism. FEBS J 274:2363–2375. doi:10.1111/j.1742-4658.2007.05771.x. 17403037

[27] Versées W, Spaepen S, Wood MDH, Leeper FJ, Vanderleyden J, Steyaert J. 2007. Molecular mechanism of allosteric substrate activation in a thiamine diphosphate-dependent decarboxylase. J Biol Chem 282:35269–35278. doi:10.1074/jbc.M706048200. 17905741

[28] Schütz A, Golbik R, Tittmann K, Svergun DI, Koch MH, Hübner G, König S. 2003. Studies on structure-function relationships of indolepyruvate decarboxylase from *Enterobacter cloacae*, a key enzyme of the indole acetic acid pathway. Eur J Biochem 270:2322–2331. doi:10.1046/j.1432-1033.2003.03602.x. 12752452

[29] Wang B, Bai Y, Fan T, Zheng X, Cai Y. 2017. Characterisation of a thiamine diphosphate-dependent α-keto acid decarboxylase from *Proteus mirabilis* JN458. Food Chem 232:19–24. doi:10.1016/j.foodchem.2017.03.164. 28490063

[30] Panda T, Gowrishankar BS. 2005. Production and applications of esterases. Appl Microbiol Biotechnol 67:160–169. doi:10.1007/s00253-004-1840-y. 15630579

[31] Joseph B, Ramteke PW, Thomas G. 2008. Cold active microbial lipases: some hot issues and recent developments. Biotechnol Adv 26:457–470. doi:10.1016/j.biotechadv.2008.05.003. 18571355

[32] Vogel C, Widmann M, Pohl M, Pleiss J. 2012. A standard numbering scheme for thiamine diphosphate-dependent decarboxylases. BMC Biochem 13:24. doi:10.1186/1471-2091-13-24. 23157214 PMC3534367

[33] Kutter S, Weiss MS, Wille G, Golbik R, Spinka M, König S. 2009. Covalently bound substrate at the regulatory site of yeast pyruvate decarboxylases triggers allosteric enzyme activation. J Biol Chem 284:12136–12144. doi:10.1074/jbc.M806228200. 19246454 PMC2673282

[34] Baker PJ, Waugh ML, Wang XG, Stillman TJ, Turnbull AP, Engel PC, Rice DW. 1997. Determinants of substrate specificity in the superfamily of amino acid dehydrogenases. Biochemistry 36:16109–16115. doi:10.1021/bi972024x. 9405044

[35] Jiang W, Bikard D, Cox D, Zhang F, Marraffini LA. 2013. RNA-guided editing of bacterial genomes using CRISPR-Cas systems. Nat Biotechnol 31:233–239. doi:10.1038/nbt.2508. 23360965 PMC3748948

[36] Ryu RJ, Patten CL. 2008. Aromatic amino acid-dependent expression of indole-3-pyruvate decarboxylase is regulated by TyrR in *Enterobacter cloacae* UW5. J Bacteriol 190:7200–7208. doi:10.1128/JB.00804-08. 18757531 PMC2580706

