# Supplementary material for: The Characterization of an Efficient Phenylpyruvate Decarboxylase KDC4427, Involved in 2-Phenylethanol and IAA Production from Bacterial Enterobacter sp. CGMCC 5087
Source: Microbiol Spectr. 2022 Apr 4;10(2):e02660-21. doi: 10.1128/spectrum.02660-21 (PMC9045302; doi:10.1128/spectrum.02660-21)
Supplement: SUPPLEMENTAL FILE 1 — Supplemental material. Download SPECTRUM02660-21_Supp_1_seq10.pdf, PDF file, 0.1 MB [file spectrum02660-21_supp_1_seq10.pdf]

TABLE S1 Strains and plasmids used in this study

Strains and Plasmids	Description	Source or reference
**Strains**		
E. coli DH5α	Φ80d*lacZ*ΔM15 Δ(*lacZY-argF*)*U169 deoR recA1 endA1 hsdR17*(r_K^- m_K^+) *supE44 thi-1 gyrA96 relA1*	TransGen
E. coli BL21(DE3)	F^- *ompT hsdS*$_B$(r_B^- m_B^-) *gal dcm* (DE3)	TransGen
Enterobacter sp. CGMCC 5087-*ΔKDC4427*	Amp^r, knockout the *KDC4427* gene	(1)
**Plasmids**		
pETDuet-1	ColE1(pBR322) ori; Amp^r [b]; P_{T7}	Novagen
pTargetF	Spc^r [a]	(2)
pETDuet-1-*KDC4427*^{E468L}	pETDuet-1 carring the *KDC4427*^{E468L} mutant	This study
pETDuet-1-*KDC4427*^{E468A}	pETDuet-1 carring the *KDC4427*^{E468A} mutant	This study
pETDuet-1-*KDC4427*^{I542L}	pETDuet-1 carring the *KDC4427*^{I542L} mutant	This study
pETDuet-1-KDC4427^{I542A}	pETDuet-1 carring the *KDC4427*^{I542A} mutant	This study
pETDuet-1-KDC4427^{A387Q}	pETDuet-1 carring the *KDC4427*^{A387Q} mutant	This study
pTargetF-*KDC4427*^{WT}	pTargetF containing the *KDC4427* wild type	This study
pTargetF-*KDC4427*^{E468L}	pTargetF containing the *KDC4427*^{E468L} mutant	This study
pTargetF-*KDC4427*^{E468A}	pTargetF containing the *KDC4427*^{E468A} mutant	This study
pTargetF-*KDC4427*^{I542L}	pTargetF containing the *KDC4427*^{I542L} mutant	This study
pTargetF-*KDC4427*^{I542A}	pTargetF containing the *KDC4427*^{I542A} mutant	This study
pTargetF-*KDC4427*^{A387Q}	pTargetF containing the *KDC4427*^{A387Q} mutant	This study

[a] Spectinomycin resistance.

[b]Ampicillin resistance.

TABLE S2 Accession numbers of *α*-keto acid decarboxylases in phylogenetic tree

***α*-keto acid decarboxylase**	**Accession number**
*Ao*PDC	XP 001819813
Aro10	KZV12623.1
*Ab*IPDC	CAA67899.1
*Ab*PPDC	gi 149242519
*Ec*IPDC	gi 118333
LlKdcA	gi 44921617
*Pm*KDC	KY441412
*Ss*PDC	gi 150951273
KDC4427	PWI79650.1

TABLE S3 Comparison of enzyme kinetics of several α-keto acid decarboxylase

**Substrates and parameters**	**KDC4427**	**Aro10 (3)**	***Ec*IPDC (4)**	***Ab*PPDC (5)**
Phenylpyruvic acid				
K_m (mM)	0.60 ± 0.02	0.10 ± 0.01	ND[a]	1.08 ± 0.09
k_{cat} (s^{-1})	186.94 ± 0.39	20 ± 2.1	ND	333.7 ± 7.8
k_{cat}/K_m ($mM^{-1}\cdot s^{-1}$)	311.76	200	ND	309
2-ketobutanoic acid				
K_m (mM)	0.66 ± 0.08	7.6 ± 0.6	ND	ND
k_{cat} (s^{-1})	185.77±3.08	3.9 ± 0.1	ND	ND
k_{cat}/K_m ($mM^{-1}\cdot s^{-1}$)	255.09	0.52	ND	ND
Indole-3-pyruvic acid				
K_m (mM)	0.015 ± 0.002	0.03 ± 0.01	0.020 ± 0.0013	0.13 ± 0.01
k_{cat} (s^{-1})	3.7 ± 0.6	5.4 ± 0.3	3.9 ± 0.007	4.1 ± 0.1
k_{cat}/K_m ($mM^{-1}\cdot s^{-1}$)	247	180	199	32
Pyruvic acid				
K_m (mM)	0.86 ± 0.15	9.7±0.1	3.38±0.18	ND
k_{cat} (s^{-1})	22.31 ± 2.79	0.34 ± 0.01	3.5±0.08	ND
k_{cat}/K_m ($mM^{-1}\cdot s^{-1}$)	26.1	0.035	1.04	ND

[a] ND means no data.

TABLE S4 Primers used in this study

**Primer**	**Sequence (5' → 3')[a]**	**Purpose**
4427-Spe I-F	AGTCCTAGGTATAAT<u>ACTAGT</u>ATGCGTACCCCATACTGCG	Amplification of *KDC4427* gene
*4427-Eco*R I-R	CAGGTCGACTCTAGA<u>GAATTC</u>TCAGGCGCTATTGCGCGCT	
$KDC4427^{I542L}$-F	CGCCGCTGCTCGGGGCGCTTACTAAAGCGCTGGAA	Construction of mutant I542L
$KDC4427^{I542L}$-R	TTCCAGCGCTTTAGTAAGCGCCCCGAGCAGCGGCG	
$KDC4427^{E468L}$-F	GAAGGGTACACGGTGCTAAGAGCGATCCACGGGCC	Construction of mutant E468L
$KDC4427^{E468L}$-R	GGCCCGTGGATCGCTCTTAGCACCGTGTACCCTTC	
$KDC4427^{I542A}$-F	CGCCGCTGCTCGGGGCGGCCACTAAAGCGCTGGAA	Construction of mutant I542A
$KDC4427^{I542A}$-R	TTCCAGCGCTTTAGTGGCCGCCCCGAGCAGCGGCG	
$KDC4427^{E468A}$-F	GAAGGGTACACGGTGGCAAGAGCGATCCACGGGCC	Construction of mutant E468A
$KDC4427^{E468A}$-R	GGCCCGTGGATCGCTCTTGCCACCGTGTACCCTTC	
$KDC4427^{A387Q}$-F	GGATCAGGGCACCTCGCAGTTCGGCGCGATCGACC	Construction of mutant A387Q
$KDC4427^{A387Q}$-R	GGTCGATCGCGCCGAACTGCGAGGTGCCCTGATCC	

[a] The underlined part is the restriction site.

REFERENCES

1. Zhang H, Cao M, Jiang X, Zou H, Wang C, Xu X, Xian M. 2014. *De-novo* synthesis of 2-phenylethanol by *Enterobacter* sp. CGMCC 5087. BMC Biotechnol 14: 30.
2. Jiang W, Bikard D, Cox D, Zhang F, Marraffini LA. 2013. RNA-guided editing of bacterial genomes using CRISPR-Cas systems. Nat Biotechnol 31: 233-239.
3. Kneen MM, Stan R, Yep A, Tyler RP, Saehuan C and McLeish MJ. 2011. Characterization of a thiamin diphosphate-dependent phenylpyruvate decarboxylase from *Saccharomyces cerevisiae*. FEBS J 278: 1842-1853.
4. Schütz A, Golbik R, Tittmann K, Svergun DI, Koch MH, Hübner G, König S. 2003. Studies on structure-function relationships of indolepyruvate decarboxylase from *Enterobacter cloacae*, a key enzyme of the indole acetic acid pathway. Eur J Biochem 270: 2322-2331.
5. Spaepen S, Versées W, Gocke D, Pohl M, Steyaert J and Vanderleyden J. 2007. Characterization of phenylpyruvate decarboxylase, involved in auxin production of *Azospirillum brasilense*. J Bacteriol 189: 7626-7633.
